# Is postgraduate leadership education a match for the wicked problems of health systems leadership? A critical systematic review

**DOI:** 10.1007/s40037-019-0517-2

**Published:** 2019-06-03

**Authors:** Betty Onyura, Sara Crann, David Tannenbaum, Mary Kay Whittaker, Stuart Murdoch, Risa Freeman

**Affiliations:** 1 Centre for Faculty Development, Faculty of Medicine, University of Toronto, St. Michael’s Hospital, Ontario, Canada; 2 Department of Psychology, University of Windsor, Ontario, Canada; 3 Department of Family and Community Medicine, University of Toronto, Ontario, Canada

**Keywords:** Leadership development, Residency, Postgraduate education, Wicked problems

## Abstract

**Purpose:**

There have been a growing number of leadership education programs for physicians. However, debates about the value and efficacy of leadership education in medicine persist, and there are calls for systematic and critical perspectives on medical leadership development. Here, we review evidence on postgraduate leadership education and discuss findings in relation to contemporary evidence on leadership education and practice.

**Method:**

We searched multiple databases for papers on postgraduate leadership development programs, published in English between 2007 and 2017. We identified 4,691 papers; 31 papers met the full inclusion criteria. Data regarding curricular content and design, learner demographics, instructional methods, and learning outcomes were abstracted and synthesized.

**Results:**

There was modest evidence for effectiveness of programs in influencing knowledge and skills gains in select domains. However, the conceptual underpinnings of the ‘leadership’ training delivered were often unclear. Contemporary theory and evidence on leadership practice was not widely incorporated in program design. Programs were almost exclusively uni-professional, focused on discrete skill development, and did not address systems-level leadership issues. Broader leadership capacity building strategies were underutilized. A new wave of longitudinal, integrated clinical and leadership programming is observed.

**Conclusions:**

Our findings raise questions about persistent preparation-practice gaps in leadership education in medicine. Leadership education needs to evolve to incorporate broader collective capacity building, as well as evidence-informed strategies for leadership development. Barriers to educational reform need to be identified and addressed as educators work to re-orientate education programs to better prepare budding physician leaders for the challenges of health system leadership.

**Electronic supplementary material:**

The online version of this article (10.1007/s40037-019-0517-2) contains supplementary material, which is available to authorized users.

## What this paper adds

The drive and mandate for leadership education for physicians continues to grow and there are calls for systematic and critical reviews of medical leadership education. This work (1) provides systematic synthesis of the educational approaches and outcomes in current postgraduate leadership education and (2) offers analytical perspectives on some of the discordance between medical leadership education and the practice demands of health systems leadership. Ultimately, it draws on contemporary theory and evidence on leadership development to present implications for practice in medical leadership education.

## Introduction

Concerns about effective leadership are gaining prominence in discourses of health system operations and transformation. And, although definitional consensus is elusive [1], it is broadly acknowledged that leadership of healthcare systems extends beyond the clinical leadership [2] activities of directing and collaborating on the treatment of patients at the point of care. Rather, health systems leadership also entails active participation in the stewardship and transformation of healthcare service delivery, resources, and policy in the public interest [1, 3, 4]. The growing emphasis on the importance of health systems leadership coincides with the extraordinary challenges facing global health systems that must contain costs while meeting public expectations for high quality care at a time of rapid technological advances, demographic shifts, and rising income inequality [1, 5]. It is in this environment that there is growing contention for physician engagement in health systems leadership given their influence over healthcare quality and resource utilization [6–9]. Indeed, there is modest, albeit mixed, empirical evidence to support a positive link between having physicians leaders in senior, structural roles, and indicators of health system effectiveness such as quality of care [10]. At the same time, evidence on physician leadership in practice suggests that health systems leadership work is fraught with challenges and paradoxical tensions. There are persistent concerns with general reluctance among physicians to engage in health systems leadership activities [11–13]. And, despite being motivated to engage in leadership in order to contribute to health system improvement, physicians leaders report feeling unable to effect system change [14, 15] and perceive health systems leadership work as costly (personally, socially, and materially) [16–18]. Tensions between physicians’ professional group (clinical) identity and leadership work demands [13, 19] are commonly reported as are relational tensions across professional and hierarchical boundaries [20, 21]. Critically, physician leaders often report feeling unprepared for their leadership roles [19, 22], and scholars have questioned whether traditional postgraduate training provides physicians with adequate education for leadership roles [7, 23].

The desire for greater physician participation in health systems leadership—and contention that better leadership education is needed—has put pressure on educators to develop leadership education programs across the continuum of medical training and practice. However, questions remain about whether leadership programs are adequately preparing upcoming physicians for the challenges they will encounter in practice. Notably, there have been critiques about investments in leadership training in healthcare, and questions about the efficacy of leadership education. These include questions as to whether leadership education in medicine can develop collective agency for reform as opposed to maintaining the status quo or entrenching medical power [24]. It also includes questions about the potentially heavily circumscribed impact of leadership development given the limited discretionary authority that leaders can have within their institutions’ policy, regulatory, and cultural constraints [25]. Furthermore, a review [26] of physician leadership development programs raised questions about the educational strategies that were utilized by extant programs. Across programs, the authors found evidence of a particularly narrow focus on individual-level (vs system) outcomes, as well as limited use of advanced leadership education tools such as interactive learning and feedback [26]. The aforementioned concerns and review findings point to a potential preparation-practice gap, whereby leadership curricula in medicine may not be designed to meet the demands of health system workplaces. Thus, it is imperative that scholars and educators update our understanding of how contemporary leadership education in medicine is functioning and whether it is evolving to better address the demands of physicians as health system leaders.

In this work, we systematically and critically examine the nature and outcomes of leadership education in medicine in light of emerging trends and evidence in education and leadership practice. Over the past decade, discussions on leadership in healthcare have started to embrace contemporary conceptualizations of leadership that define leadership as mutually influencing, power-sharing, and collective [27, 28]. Theoretical conceptualizations in this area include terms such as ‘shared,’ ‘collective,’ ‘complex adaptive’ and ‘relational’ leadership among others [28]. Notwithstanding conceptual distinctions among these approaches, they all have at their core a reframing of leadership as exercised within groups along relational lines that are not necessarily defined by formal roles or traditional hierarchies [28]. Further, they attend to the socio-political and material complexity that has led many health system challenges to be characterized as ‘wicked problems’—problems that are dynamic, and multi-faceted, with conflicting demands that are recalcitrant to linear resolution [29]. These contemporary conceptualizations of leadership may necessitate different pedagogical approaches to leadership development. Thus, it is important to examine whether these newer leadership discourses are influencing the landscape of leadership education in medicine, as well as study how they may be influencing program outcomes.

A critical systematic review, such as this one, provides the opportunity to discuss patterns, trends, and outcomes of leadership education in relation to a concrete synthesis of evidence about extant programs. Here, we focus on synthesizing evidence on the characteristics and efficacy of postgraduate leadership development programs. Ultimately, we aim to inform critical discussion on the current landscape of leadership education in medicine in relation to contemporary evidence on leadership development in addition to exploring potential preparation-practice gaps for physician leaders.

## Methods

This study was part of a larger program of inquiry that included a state-of-the-art review on research about physician health systems leadership and a formative evaluation study on the development of new leadership development programs.

### Search strategies, screening, and selection methods

Initially, we commissioned two broad searches on physician leadership in health systems. Two research librarians independently conducted title and abstract keyword searches across multiple databases (ABI/Inform Global, CINAHL, CBCA Education, Education Source, EmBASE, ERIC (ProQuest), OVID Healthstar, OVID MedLine, PsycINFO, Scopus, and Social Science Abstracts). These searches yielded a total of 4,124 articles. A third librarian-conducted search of the same databases targeted leadership education programs specifically (see Appendix A of the online Supplementary Material for the unique search terms used in each search). This third search yielded 551 articles. We also manually reviewed citations, and reference lists of papers that underwent full-text review to find articles that may not have been captured through the database search. This search yielded an additional 16 articles. Overall, 4,691 articles were identified.

We applied standard systematic review procedures for sifting abstracts, examining full papers, and abstracting data. Specifically, the first and second author screened all identified abstracts for eligibility criteria and identified 220 abstracts for full-text review. Articles underwent full-text review if they met the following inclusion criteria: 1) published in English between 2007–2017 in a peer-reviewed journal, and 2) the primary purpose of the article was the description or evaluation of a leadership development program (i.e., an educational intervention that explicitly targeted leadership training for postgraduate medical trainees/residents). Articles were excluded if they 1) included comparative studies, descriptions of multiple programs, or 2) focused exclusively on leadership within a narrowly defined clinical area (e.g., leadership in trauma resuscitation). EPPI Reviewer 4 software was used as a review management tool. Ultimately 31 papers met all eligibility criteria.

### Data abstraction, analysis, and synthesis

We developed a structured abstraction tool to facilitate extraction of key information from reviewed articles. Our abstraction categories are similar to Frich et al. [26] (focusing on education setting, targeted learners, teaching methods, education content and outcomes). However, we also abstracted data on expressed guiding frameworks/theories that informed program design. Like Frich et al. [26], we used an adapted version of Kirkpatrick’s model of learning outcomes [30] to classify abstracted outcome data (Tab. 1). We employed a dual-abstraction strategy[BO1] and [SC] abstracted data upon an initial read and a second team member reviewed abstracted data for accuracy. Discrepancies were noted and reconciled following a third review of the article in question by [BO] and [SC]. We used the Medical Education Research Quality Instrument (MERSQI) [31] to rate the quality of all quantitative evaluation studies/components. We included four program descriptions without evaluation components to provide comprehensive coverage of available leadership programming in the peer-reviewed literature.

**Table 1:** Levels of educational outcomes from Kirkpatrick studies

Level	Outcome	Description
1a	Learner reaction	Learners’ reactions to program (e.g., satisfaction with learning experience)
2a	Attitudinal changes	Changes in learners’ attitudes and beliefs
2b	Knowledge or skill acquisition	Acquisition of knowledge (e.g., concepts or principles), and/or skills
3a	Behavioural change	Application of knowledge/skills in an educational or practice setting
3b	Learner achievements	Learner academic and professional achievements (publications, presentations, awards, professional positions)
4	Organizational or health systems change	Changes in work units and institutions or in the operation and organization of health systems

## Results

Review findings are presented in tabular and narrative form. The 31 identified studies represented five countries across three continents. Overviews of program features and content are presented in Tab. 2 and Tab. 3 (in the online Supplementary Material); comprehensive details about individual programs are presented in Tab. 4, which can be found in the online Supplementary Material. The average MERSQI score across two raters for the 18 quantitative studies/study components was 8.4 (range 6 to 12.5). Kappa co-efficient for inter-rater reliability was 0.94 (almost perfect agreement between raters).

**Table 2:** Overview of central features of postgraduate leadership programs in medicine (*n* = 31)

Feature	No. (%)
**Country**	
US [2, 8, 33, 36–41, 43, 44, 52, 54–56, 76, 81, 82]	18 (58)
UK [23, 32, 45–47, 50, 51]	7 (22.6)
Canada [48, 53]	2 (6.5)
Australia [34, 42]	2 (6.5)
Germany [35]	1 (3.2)
Netherlands [49]	1 (3.2)
**Education setting/program type**	
Formal integrated residency program [32, 33, 40, 44, 51, 54, 76, 82]	8 (25.8)
University-based course/elective programs [8, 35, 37, 39, 41, 43, 47–49, 52, 55]	11 (35.9)
Hospital/academic medical centre-based elective programs [2, 36, 38, 46, 53, 56]	6 (19.4)
State or nationally organized curricula [23, 34, 42, 50]	4 (12.9)
Medical society/association program [81]	1 (3.2)
Unclear or unspecified [45]	1 (3.2)
**Targeted learners/entry level**	
PGY1 [32, 33, 37, 43, 76]	5 (16.1)
PGY2 [2, 54]	2 (6.5)
PGY3 [45, 53]	2 (6.5)
PGY4 [51]	1 (3.2)
Multiple PG levels [35, 40, 42, 44, 48, 49, 52, 55]	8 (25.8)
Chief residents [8, 36, 38, 41]	4 (12.9)
Senior residents and fellows [39]	1 (3.2)
Residents (PGY4) and senior physicians [34]	1 (3.2)
Medical students residents and fellows [81]	1 (3.2)
Level not specified [23, 46, 47, 50, 56, 82]	6 (19.4)
**Targeted learners/specialties**	
Internal medicine [2, 43]	2 (6.5)
Multiple specialties [23, 34–36, 38, 41, 42, 44, 46, 49, 56, 76]	12 (38.7)
Multiple secondary care specialties [32]	1 (3.2)
Paediatrics [40]	1 (3.2)
Paediatric anaesthesia [37]	1 (3.2)
Pathology [39]	1 (3.2)
Primary care (general practice/family medicine) residents [33, 45, 47, 51, 54]	5 (16.1)
Psychiatry [48]	1 (3.2)
Radiology [52, 55]	2 (6.5)
Neurosurgery [8]	1 (3.2)
Surgery [53, 82]	2 (6.5)
Unclear/unspecified [50, 81]	2 (6.5)
^a^Structured multi-professional/management-clinician learning components [32, 44, 82]	(3/31)
**Duration**	
Short-term interventions (1 day–1 month) [2, 35, 36, 38, 41, 53, 55]	7 (22.6)
Medium-term interventions (intermittent sessions or >1 month <1 year) [8, 39, 43, 47, 48, 52, 81]	7 (22.6)
Longitudinal interventions (intensive rotations or ≥1 year programs) [23, 32, 33, 37, 40, 42, 44, 49–51, 54, 56, 76, 82]	14 (45.2)
Length varies [34, 46]	2 (6.5)
Unknown/not specified [45]	1 (3.2)
**Credential**	
Certificate in Healthcare Management and Leadership [76]	1 (3.2)
Accredited postgraduate certificate [50]	1 (3.2)
Diploma in Health and Public Leadership [32]	1 (3.2)
MSc in Health and Public Leadership [32]	1 (3.2)
Masters in Public Health [33, 56]	2 (6.5)
None/not specified [2, 8, 23, 34–49, 51–55, 81, 82]	26 (83.9)

^a^Overlaps with other categories above

### Targeted learners

Postgraduate leadership programs were almost exclusively uni-professional; only 9.7% (3/31) programs offered a structured multi-professional or joint management-clinician learning components. Further, 51.6% (16/31) of programs targeted residents from a single specialty or subspecialty whereas 38.7% (12/31) were open to learners across multiple primary and secondary care residency programs. Leadership development targeted uniquely toward primary care generalist physicians (including internal medicine) represented only 22.6% (7/31) of programs while specialized/secondary care programs represented 32.3% (10/31) of programs. Details on entry-level requirements for program admission are outlined in Tab. 2.

### Program duration and credentials

Programs ranged in structure and duration from 1‑day workshops to 4‑year integrated residency programs, which we group into three categories in Tab. 2: short-term interventions (1 day to 1 month programs); medium-term interventions (intermittent sessions or >1 month to <1 year); and longitudinal programs (intensive continuous rotations or ≥1 year). Most longitudinal programs were formal, academic, postgraduate medical programs that integrated clinical and leadership training in their curricula in some form. The majority of programs did not offer unique academic credentials to graduating learners (Tab. 2).

### Conceptual foundations of leadership education

As highlighted in Tab. 3 of the online Supplementary Material, 29% (9/31) of programs did not report the conceptual (theoretical or competency-based) underpinnings for their leadership programming. Approximately 50% of programs were informed by the competency models of regulatory associations (e.g., CanMEDS, Medical Leadership Competency Framework, MLCF). Only 19.4% (6/31) of programs were informed by a specified theoretical model of leadership including shared leadership [32], adaptive and collective leadership [33], and relational leadership [34] and transformational leadership [35]. It is important to note that MLCF is informed by shared leadership theory.

### Content areas for leadership education

Over 75 topic areas were covered across programs (Tab. 3 of the online Supplementary Material). Three of the most common topic areas across programs were quality improvement and patient safety (48.4%, 15/31), conflict management and resolution (35.5%, 11/31), and financial management (25.6%, 8/31). ‘Leadership’ was identified as a broad curriculum topic area across many programs (32.3%, 10/31) often without specification of the conceptual or theoretical underpinnings of the training delivered. Few programs explicitly covered health systems leadership (19.4%, 6/31) or other macro-level health systems topics such as policy (19.4%, 6/31), economics (12.9%, 4/31), and equity (3.2%, 1/31).

### Teaching and assessment approaches

Most programs employed multiple educational strategies (Tab. 3 of the online Supplementary Material). Common educational approaches across programs included lectures/workshops with didactic and interactive components (74.2%, 23/31), assigned project work (38.7%, 12/31) and case-based learning (22.6%, 7/31). Longitudinal programs were more likely to adopt leadership development approaches such as experiential placements (8/14 programs), mentorship (5/14 programs), and coaching (3/14 programs) than shorter- and medium-term programs (experiential placements, 1/17 programs; mentorship, 2/17 programs; and coaching, 2/17 programs).

Although most programs reported employing some form of learner assessment *or *feedback (Tab. 3 of the online Supplementary Material), few programs provided learners with feedback on actual (22.6%, 7/31) or simulated (6.5%, 2/31) leadership performance. Further, only 32.3% (10/31) of programs used objective forms of assessments such as tests.

### Learning outcomes

Over 55% of programs reported positive learner reactions (Kirkpatrick Level 1) to leadership education [2, 32, 36–49]. Attitudinal shifts (Level 2A) were reported for a few programs; they included changes to career aspirations [50] and increased interest in pursuing leadership [2] and quality improvement/patient safety roles [44], enhanced engagement and motivation [51], enhanced confidence [38, 41, 51, 52] and changed (positively) values about the role of clinical leadership [50]. Several programs also reported enhanced knowledge and skill (Level 2B) outcomes across intra-personal, interpersonal, and technical domains. For example, some programs reported personal growth among participants with regard to increased self-awareness [2, 45, 47, 49, 50] and stress management [53]. Knowledge gains were reported in several areas including awareness of organizational contexts [47, 49, 50] and system functioning [32, 42], leadership theory [2, 38], and financial management [32, 53, 54]. It is noteworthy than one of the studies reported finding no significant knowledge gains between the leadership intervention group and a control group—despite differences in externally evaluated leadership skills [35].

Across programs there were reports of gains in multiple skill domains, including general/unspecified leadership/management skills [36, 39–41, 45, 51, 54]. More specifically, learners were reported to gain skills in the following domains: team work [32, 50, 55], clinical leadership [2, 37], transactional and transformation leadership [35], change management and service improvement [50], teaching [36, 40, 41], interpersonal skills [2], assertiveness [51], practice management [37, 53], conflict resolution [37, 41, 53], time management [47, 51], group facilitation [41, 51], inter-specialty collaboration [41], negotiation [32, 54], networking [32, 42, 47], relationship building [42, 47], strategic thinking and planning [32, 54], project management [47], goal setting [32], communication [47, 55], feedback delivery [41, 53] and quality improvement skills [44, 46, 54], among others. See Tab. 4, of the online Supplementary Material, for a comprehensive overview of outcomes for each program.

A small number of programs reported outcomes related to behavioural change and learner accomplishments (Levels 3A and B). These outcomes included improved interactions with hospital administrative authorities [55], successful employment [42, 56], securement of leadership roles such as chairs and committee representatives [32, 55], and personal achievements such as publications, presentations, and awards [32, 40, 46]. A few programs also reported outcomes related to organizational and system changes (Levels 4A and 4B) in places where learners undertook projects or experiential placements. For example, several studies [32, 40, 42, 46, 47] reported that learners had a positive impact on enacting healthcare service improvements in several areas (e.g., reduced waiting times, waste reduction, reduced error). In other examples, postgraduate trainees were reported to have positive impacts on group dynamics [55], inter-organizational communication [42, 47], and staff hiring practices such as enhancing diversity [40]. One study reported that learners were instrumental in championing new legislative policies [40]. A separate study reported positive shifts in health system stakeholders’ attitudes regarding their belief in trainees’ abilities to produce change [50].

## Discussion

Our review found modest evidence that extant postgraduate leadership education can generate positive, individual-level outcomes (e.g., skill gains) and that trained residents can contribute effectively to health service improvements. However, like earlier work reviewing physician leadership development more broadly, [26] we found that the scope of leadership education programs in postgraduate medicine remains relatively narrow, and largely focused on individual-level development. Similarly, our findings also highlight the limited range in leadership development strategies that are employed in postgraduate programs. Whereas our findings raise some concerns that the academic preparation-practice gaps alluded to by Frich et al. [26] may be persistent, our review identifies new, modest trends toward the inclusion of broader perspectives on leadership. Ergo, in the ensuing discussion we draw attention to key observations and discuss the relation of preparation-practice gaps to existing evidence about leadership education and health systems leadership.

First, there remains a prevailing programmatic emphasis on individual leader competence versus leadership development^1^ that is focused on capacity building for a collective (e.g., teams, institution)—with attention to collective outcomes [57]. Whereas individual leader competence is undoubtedly necessary, leadership scholars argue that individual-level competence is insufficient for effective health systems leadership where the challenges faced are often too intractable for traditional individualist/heroic approaches to leadership [29, 58]. As noted earlier, Rittel and Webber’s [59] typology of critical, tame, and wicked problems is a useful one in thinking about leadership [60]. Critical problems in medicine (e.g., an acute heart attack) demand swift action leaving little time for uncertainty or procedure; tame problems (e.g., elective heart surgery) may be complicated puzzles but they are manageable with relatively linear tools and are likely to have been resolved before [60]. Wicked problems, however, are intertwined with deeply complex, social and cultural issues; they are situated across institutions and may have no clear ‘stopping points’ at which the problem could be said to be solved (e.g., developing equitable arrangements for providing government funded services to ageing populations at a time of increasing medical ability to maintain life) [29, 60]. Individual leader development may suffice to prepare future leaders to navigate critical and tame problems that manifest in acute clinical microsystems but be inadequate in preparing physicians—individually and as a collective—to engage in the leadership activity required to address the wicked problems of contemporary health systems. Addressing wicked problems, Grint argues, requires a reframing of leadership as ‘influencing a collective to take joint responsibility for collective problems’ [60]. Contemporary paradigms of leadership that espouse shared, process-oriented, mutually influential views of leadership places more value on developing collective capacity for leadership than traditional models that focus on skill enhancement [61]. However, only a few programs reported being explicitly informed by these contemporary views of leadership.

We must note here that physician participation in *both* traditional, individualist/heroic leadership practices (e.g., transformational/transactional leadership) [62] *and* contemporary boundary-spanning, collaborative leadership practices has been linked to the successful stewarding of health system reforms [14, 63, 64]. Thus, the argument for integrating more contemporary, collectivist views of leadership in leadership education is not wedded to a binary perspective of heroic vs post-heroic leadership [65]. Rather it is an argument that educating residents to understand and enact these types of approaches better prepares them for the demands of leadership. In practice, managing health system problems (such as implementing reform) demands hybridized or interwoven approaches where heroism and collectivism can coexist as demanded by shifting contexts and circumstance [65, 66]. That notwithstanding, leadership development in medicine is heavily weighted in the individualist realm. Thus, the inculcation of newer, collective conceptions of leadership would require leadership development approaches that work to shift mindsets about the primacy of individual leaders in addressing health system problems [67]. Tackling wicked problems demands forms of influence that cannot be conferred simply by occupying senior roles. Others cannot be forced to follow you, they must have the volition to help [60] and knowledge workers in modern health systems are disinclined to enact top-down visions [27]. Leading in such environments involves facilitating contexts in which others are also willing and able to lead to address shared problems [58] within the scope of their expertise. It thus behoves medical educators to be clear and balanced about the models of leadership they are promoting and ensure that curricula are strategically aligned with the underlying program aims.

Second, our findings show that although almost half of all residency leadership programs covered quality improvement and patient safety, fewer than 15% covered content domains explicitly related to system-level leadership (e.g., policy, equity, systems thinking). Given that most physicians operate within clinical microsystems—‘small, functional units at the front-lines of delivering care to patients’ [68]—it is important that emerging leaders are equipped to manage clinical microsystems. However, as patients move across multiple clinical microsystems in their life journeys it is important that those in leadership roles understand the macro-level issues that influence the success of inter-dependent clinical microsystems. Extant research tells us that leaders in general operate across vast knowledge and practice domains—often requiring a breadth of both professional-industry specific and general-administrative knowledge and skill [69]. It is argued that true mastery in leadership means the ability to cross and bridge differing domains—becoming an expert in a mega-domain with many subsets [69]. With this view, health systems leadership is a significantly broader domain of practice than clinical leadership and the skills acquired during clinical leadership training may not be transferrable to health systems leadership environments without concerted effort to develop those skills for that context. Evidence on developing adaptive expertise in clinical reasoning shows that contextual variation (seeing concepts in multiple contexts) is critical to building the cognitive store of exemplars necessary for expertise, as well as for preparing students for the transfer of learning to practice [70]. As our understanding of expertise in other domains is also relevant to leadership education [69], leadership programs should strive to provide learners with opportunities to learn across multiple contexts.

Third, like Frich et al. [26] our findings suggest that key leadership development tools such as experiential learning, mentoring, coaching, and feedback remain underutilized in postgraduate medical education. This runs counter to evidence on leadership development which demonstrates that challenging, practical experiences are powerful learning tools that function optimally when complemented by feedback on learning progress [71]. Although learning from experience is not guaranteed, residents within leadership programs may be particularly suited to benefit from such placements because they are likely to be in *learning mode* [72] during such placements (i.e., they are positioned to frame and pursue their placement experience with an orientation toward growth and learning) [72]. Promisingly, approximately 60% of longitudinal programs did report using experiential placements. Shorter-term programs should strive to develop alternative strategies that provide workplace-based exposure to leadership in practice (e.g., shadowing, rounds).

Further, leadership education remains exclusively uni-professional, and generally targeted toward single specialities. This persistent uni-professionalism raises concerns about whether budding physician leaders are prepared enough for the multi-professional and inter-sectoral relationship building that will be demanded of them in practice. The expansion of boundary-crossing social networks and social capital has been identified as an important feature of successful leadership development [57, 73]. Successful leadership has been linked to the quality of the social network in which a leader is embedded [73]. It may be particularly important for enhancing collective leadership capacity given the distributed nature of health systems whereby leadership activity must be spread across organizations/units in order to adequately respond to challenges that appear at different units of governance [74]. Indeed, research on physicians leadership in practice suggests that the absence of relationships among leaders across specialities and across clinical/non-clinical boundaries [14] impedes leaders’ efforts to enact health service improvements. On the other hand, a recent critical examination of inter-professional education research and practice [75] suggests that uni-professional educational environments may be more efficacious for teaching collaborative skills than inter-professional educational settings—if coupled with workplace-based interventions. However, gains in collaborative skills may not translate into social capital across a given system without dedicated opportunities for relationship building. It is important for educators to design curricula strategies that support the development of social capital across professional boundaries.

Promisingly, our findings present modest evidence of an emergent new wave of residency leadership interventions where leadership education is formally integrated into a broader residency curriculum, giving learners ongoing exposure to both clinical skills training and leadership development experiences. Integration of leadership education into clinical practice training may also encourage physicians to view leadership work as part of their regular professional practice, thus minimizing perceived commonly experienced tensions between clinical and leadership identities [13]. Many of these ‘new wave’ programs [32, 33, 40, 50, 56, 76] were also more likely to have curricula content on macro level health systems issues, and explicitly strive to enhance physician engagement in health systems leadership. Higher quality evaluative studies are needed to ascertain the impact of these types of programs on both individual-level and collective outcomes.

In summary, our review highlights preparation-practice gaps in leadership development and integrates knowledge on evidence-informed educational strategies that may be useful in addressing these gaps. Furthermore, we contend that alternative pedagogies may be needed to instil the value of contemporary, collectivist approaches to leadership. Specifically, it may be useful to incorporate approaches that go beyond predominant paradigms of education [77] (that are generally focused on skill acquisition and assimilation). For example, in leadership programs where the goal is to develop leaders’ change agency for system reform, approaches such as transformative learning [78, 79] would be appropriate as they encourage learners to critically reflect on and address factors that contribute to the maintenance of the status quo [80].

As noted earlier, previous scholarship has identified similar concerns about potential preparation-practice gaps in extant leadership education. The persistence of these gaps raises questions about the difficulties inherent in transforming established educational cultures. These challenges may have to do with resource constraints as well as inadequate knowledge mobilization and faculty development. Deeply rooted cultural change resistance may also be at play; senior educators/leaders may inadvertently reproduce antiquated frames of reference about leadership that may no longer be appropriate for today’s health system challenges [13]. Furthermore, contemporary or evidence-informed approaches to leadership education and practice may be at odds with existing ways of organizing across health systems that are still largely structured hierarchically. Deeper structural and policy changes may be required to better address health systems wicked problems, and to promote needed cultural and evidence-informed shifts in leadership development in medicine. Encouragingly, our findings on newer programs show subtle but promising movements in the structure and content of leadership programming for residents.

### Strengths and limitations

Our work is limited to peer-reviewed literature and some leadership development programs may be excluded from this review as a result. Furthermore, the quality of evaluation across programs was generally poor, and authors may have omitted descriptive details about their interventions. Consequently, our analysis of these programs may be incomplete. Nevertheless, the work draws on a rigorous empirical synthesis of data on leadership programming to provide a critical, evidence-informed, discussion on how the scope of leadership development in medicine needs to change in order to better prepare physicians for the demands of health systems leadership.

## Caption Electronic Supplementary Material

Appendix A. Search terms and strategies

Table 4 Details of Studies Examining Leadership Education Programs for Medical Residents

Summary of postgraduate leadership programs’ guiding frameworks, curricular content, instructional approaches, and evaluation designs (*N* = 31)

## Footnotes

1 Leadership development is often in common usage as an umbrella term for both individual leader development and the broader collective leadership development.
