# Supplementary material for: Is postgraduate leadership education a match for the wicked problems of health systems leadership? A critical systematic review
Source: Perspect Med Educ. 2019 Jun 3;8(3):133–42. doi: 10.1007/s40037-019-0517-2 (PMC6565666; doi:10.1007/s40037-019-0517-2)
Supplement: Supplementary file 1 — Appendix A. Search terms and strategies [file 40037_2019_517_MOESM1_ESM.docx]

Appendix A. Search terms and strategies

Initial title and abstract search conducted by institutional librarian using search terms “leader AND (health* system* OR health services system* OR health care system*) yielded 1147 articles

Second title and abstract search conducted by second institutional librarian using search terms “physicians AND leader* (AND competenc* OR framework* OR capabilities OR skill*)” yielded 2977 articles

Third title and abstract search conducted by second institutional librarian using search terms “physicians AND (leadership development OR leadership program OR leadership curriculum)” yielded 551 articles

4675 articles for review of title and abstract

Manual search of reference lists yielded 16 articles for review of title/abstract

4455 articles excluded

220 articles identified for full-text review
