# Supplementary material for: Is postgraduate leadership education a match for the wicked problems of health systems leadership? A critical systematic review
Source: Perspect Med Educ. 2019 Jun 3;8(3):133–42. doi: 10.1007/s40037-019-0517-2 (PMC6565666; doi:10.1007/s40037-019-0517-2)
Supplement: Supplementary file 2 — Table 4 Details of Studies Examining Leadership Education Programs for Medical Residents [file 40037_2019_517_MOESM2_ESM.docx]

| Supplementary Material: Table 4 *Details of Studies Examining Leadership Education Programs for Medical Resident*s |
| --- |

| **Source (first author, year, reference)** | **Program Type, Country** | **Targeted Learners** | **Program description & credential** | **Curricular activities & Guiding frameworks** | **Curricular content:**  ***Topics covered, Assessment and Feedback*** | **MERSQI for quantitative components** | **Outcomes^^[[1]](#footnote-1)^^** | **Program evaluation methods & Reported Outcomes**  **(Kirkpatrick levels in brackets)** |
| --- | --- | --- | --- | --- | --- | --- | --- | --- |
| 1. Ackerly et al., 2011 | Formal integrated residency program, US | Residents from multiple specialties with previous graduate management training or several years of management experience  Entry at PGY1 | Integrated into core residency curriculum; extends 3-year residency to 4 years  ***Credential***:  Certificate in health care management and leadership | - Didactic component - Experiential management rotations under senior manager guidance - Longitudinal project on high priority initiative - Mentorship   ***Guiding frameworks***: N/A | - Health system management & operations - Financial management & planning - Quality improvement & safety - Information technology/informatics - Global strategy and program development - Research enterprise management - Clinical service enterprise management - Supply chain management   **Assessment & Feedback**  None specified. | N/A | N/A | No evaluation data. |
| 2. Agius et al., 2013 | Formal integrated residency program, UK | Residents in multiple secondary care specialties  (Combined learning opportunities with General Management Trainees (GMT) in the NHS)  Entry at PGY1 | Integrated into 4-year core residency curriculum; comprises 50% of residents’ time  ***Credential:***  PgDip/MSc in Health and Public Leadership | - Action learning sets - Reflection - Experiential learning modules - Personal study - Project (workplace-based projects) - Shadowing   ***Guiding frameworks:*** Shared Leadership; MLCF^^[[2]](#footnote-2)^^ | - Fundamentals of health and public leadership - Health and public policy - Organizational development & leadership - Governance and performance - Future challenges of health and public leadership   **Assessment & Feedback**  Reflection tool designed for formative feedback  Peer feedback | N/A | 1  2A,B  3A,B  4 | ***Methods:*** Qualitative  ***Analytical approaches:*** Framework thematic analysis  ***Data sources:*** interviews + program documentation (i.e., progress reports, year-end reviews, annual development plans)  ***Outcomes:***   - Residents perceived program positively (1) - Enhanced attitudinal qualities including self-efficacy about leadership, confidence, assertiveness, personal agency, and system awareness (2A) - Enhanced team work and collaborative skills (2B) - Enhanced ability to initiate service change/improvements and establish priorities (2B) - Demonstrated enhanced skills in diplomacy and negotiation, networking, strategic thinking, decisiveness and objective setting; improved technical understanding (e.g., finance) as reported by evaluators (3A) - Program participants’ received award, published, and held leadership roles (chairs, committee reps) (3B) - Service improvements via projects (4) |
| 3.Bircher et al., 2013 | Formal integrated residency program, UK | Primary care/general practice residents  Entry at Year 4 | Extends 3-year residency to 5 years  ***Credential:***  None specified | - Experiential work-place placements with project-leadership opportunities; - Sessions/lectures (didactic and interactive) - Online discussions in virtual learning environment - Role-play   ***Guiding frameworks:*** MLCF | - Assertiveness - Time management and procrastination - Motivation theory and engaging others - Difficult conversations - Management (services) - Management (others, performance - Chairing meetings and minute taking - Business management - Quality Improvement - Health system (NHS) structure and finances   **Assessment & Feedback**   - Feedback from program director on chaired meetings | N/A | 2A,B | ***Methods****:* Evaluation methods not provided. Qualitative feedback was solicited from residents and reported  ***Outcomes:***   - Improved confidence, assertiveness, motivation, and engagement, improved awareness of system functioning and change management processes (2A) - Improved time management, group facilitation skills (2B) |
| 4. Blumenthal et al., 2014 | Hospital-based elective program, US | Internal medicine residents in Year 2 | Pilot program offered during month-long ambulatory care rotations; 2-3 hours per week for 4 weeks  ***Credential:***  None specified | - Group discussions (focused on case studies, videos about physician leadership, role plays); - Small group meetings with reading assignments and case studies   ***Guiding frameworks:*** N/A | - Clinical leadership - Leadership styles - Authentic leadership - Emotional intelligence - Leading clinical teams - Model of effective leadership   **Assessment & Feedback**  None specified. | 8.5 | 1  2A,B | ***Methods:*** Mixed method  Post-program survey with Likert scale and open-ended questions  ***Outcomes:***   - Positive perception of content and structure (1) - Increased understanding of strengths/weaknesses as a leader (2A) - Increased interest in pursuing leadership opportunities (2A) - Increased knowledge of leadership styles (2B) - Improved ability to address interpersonal challenges (2B) - Improved clinical leadership skills (2B) |
| 5. Brandon & Mullan, 2013 | University-based elective program, US | Radiology residents (all cohort levels) and fellows | 7 modules (90 minutes each) across 1 year  ***Credential:***  None specified | - Lectures (didactic) - Interactive large-group analysis - Case-based small-group learning   ***Guiding frameworks:***  N/A | - Finances (costing analysis) - Quality improvement - Practice management (groups and compensation) - Group governance and mergers - Health care policy and economics - Negotiation - Conflict management   ***Assessment and Feedback:***  Formative assessment including multiple-choice and true/false questions | 10 | 2A,B | ***Methods:*** Pre-post evaluation (self-report/ tests)  ***Outcomes:***   - Enhanced confidence across all modules (2A) - Enhanced knowledge (tested) (2B) |
| 6. Cerrone et al., 2016 | Hospital-based elective program, US | Incoming chief residents in multiple specialties | 1 day (9 hours)  ***Credential:***  None specified | - Lectures (didactic) - Panel of current CRs sharing experiences and best practices - Objective Structured Teaching Encounters (OSTE)   ***Guiding frameworks:*** Emotional intelligence (EI) theory | - Leadership responsibilities in CR role - Communication skills - Interpersonal skills - Delivering difficult feedback - Interpersonal conflict resolution   **Assessment & Feedback**   - TalentSmart® EI inventory pre-program with scores and improvement strategies - OSTE with peer-feedback | N/A | 1  2B  3B | ***Methods:*** Post-program survey; OSTE scores  ***Outcomes:***   - Positive reaction to program experience (1) - Improved leadership skills (2B) - Self-reported skill development expected to lead to behavioural change and organizational impact (3B) - Improved OSTE scores over multiple OSTEs (3B) |
| 7. Edler et al., 2010 | University-based elective residency fellowship, US | Pediatric anesthesia  residents in  Year 1 | 1-year program  ***Credential:***  None specified | - Progressive experiential projects (low-cost to independent medical management) - Experiential learning (team leader /member roles)   ***Guiding frameworks:***  US military leadership curriculum | - Decision making (proactive) - Collaboration, and cooperation, - Conflict resolution - Planning and program design - Technical planning - Human factors - Organizational Culture - Quality Improvement   ***Assessment and Feedback:***   - Feedback from program staff and hospital leadership - Self-assessment through semi-annual self-evaluation forms | 9.5 | 1  2B  3A,B | ***Methods:*** Mixed-method (faculty quantitative and qualitative evaluation of resident clinical judgment; resident interviews)  ***Outcomes:***   - General satisfaction with program (1) - Improved practice management and conflict resolution skills (2B) - Improvements in clinical and leadership judgment scores as rated by faculty (2B, 3A) - Residents performed ongoing leadership roles (3B) |
| 8. Eubank et al., 2012 | Formal integrated preventive medicine residency program, US | Family medicine/ preventative medicine residents  Entry at PGY1 | Integrated into core residency curriculum - 4 years and 6 months or 5 years depending on start of electives  ***Credential:***  MPH | - Seminars/workshops - Course work (masters level) - Experiential practicum/Improvement project - Coaching   ***Guiding frameworks:*** Adaptive leadership, collective leadership, | - Team performance - Systems thinking - Narrative - Emotional awareness and reframing - Personality types - Positive power and influence - Situational leadership - Conflict resolution - Negotiation - Change management - Adaptive leadership competencies - Quality Improvement (statistical measurement, continual improvement) - Strategic management of Health Care Institutions - Financial Management - Design and Improvement of Clinical Microsystems   ***Assessment and Feedback:***   - Faculty surveys, direct observation, and committee reviews to evaluate   progress and practicum implementation | N/A | N/A | No evaluation data |
| 9. Farver et al., 2016 | Hospital-based elective program, US | New chief residents in multiple specialties | 2-day workshop  ***Credential:***  None specified | - Workshops - Online learning academy   ***Guiding frameworks:*** Emotional intelligence model | - Emotional intelligence - Self-awareness - Time management - Systems thinking - Leadership in health care (broad ) - Physician health - How to Mentor - Cultural competence - Learning organization - Teamwork/team building - Organizational culture and models - Value-based health care - Professionalism - Effective communication - Conflict resolution - Quality in health care - Continuous improvement in health care - Change implementation   ***Assessment and Feedback:*** None specified. | 8.5 | 1  2A  2B | ***Methods:*** Pre-post self-report survey  ***Outcomes:***   - Perceived program effectiveness (1) - Increased comfort level with leadership skills (2A) - Improved knowledge/familiarity with leadership concepts (2B) |
| 10. Foster et al., 2008 | Academic medical center-based elective program, US | Residents in multiple specialties –  Cohort level not specified | Integrates 2 years of  leadership preventive medicine (LPM)  training with another core residency program  ***Credential:***  MPH | - Experiential clinical leadership rotations - Online learning portfolio - Experiential public health placements - Mentorship - Coaching - Seminars (didactic) - Work rounds for progress updates and multidisciplinary interaction - Journal club - MPH coursework   ***Guiding frameworks:*** ACGME^^[[3]](#footnote-3)^^ competencies | - Clinical microsystems and local public health systems - Public health and critical issues in health - Preventive medicine - Quality Improvement (continuous improvement, statistics) - Medical culture - Leadership development - Writing for publication   ***Assessment and Feedback:***   - Feedback from faculty, coaches, peers, improvement specialist during work rounds - Feedback from faculty, coaches, and program director informed by online portfolio - Self-assessment | N/A | 3B | ***Methods:*** None provided.  ***Outcomes:***   - Residents successfully secured employment and sought further training opportunities (3B) |
| 11. Hanna et al., 2012 | Academic medical center-based elective program, Canada | Surgery residents –  PGY3 + | Annual single-day workshop  ***Credential:***  Not specified | - Lectures (interactive) - Case-based learning - Roleplay - Scenario simulation models - Small-group problem-solving sessions   ***Guiding frameworks:*** N/A | - Giving feedback - Delegating duties - Teamwork - Time management - Making rounds - Stress management - Effective learning while on services - Teaching at bedside and in operating room - Conflict management - Negotiating employment - Hedging risk - Financial management - Practice (private) management   ***Assessment and Feedback:***   - Live feedback on simulated activity | 9 | 2A,B | ***Methods*:** Pre-post survey (self-assessment)  ***Outcomes****:*   - Significant improvements in managerial skills incl. giving feedback, effective learning, and teaching (2B) - Significant improvements in residents’ perceived preparedness to perform certain duties including negotiating employment, manage finances, hedging malpractice risk, and managing a private practice (2A,B) |
| 12. Heitkamp et al., 2017 | University-based elective program, US | Radiology residents – entry at any point during residency | Weekly meetings held every 2 weeks – participation length varies  ***Credential:***  None specified | - Group discussions (health care literature) - Personal study (leadership books) - Local and national leadership opportunities - Independent research projects   ***Guiding frameworks:*** N/A | Topics vary; typically related to:   - Health care economics, - Organizational leadership - Business of radiology, Health policy - Practice management.   ***Assessment and Feedback:***  None specified. | 7 | 2A,B  3A,B | ***Methods*:** Post-program self-report survey administered when participants had graduated and were in practice  ***Outcomes****:*   - Improved career preparedness (2A) - Participants assumed leadership roles (3B) - General improvements across areas of practice including communication, interacting with referring physicians, career development, understanding the business of radiology, team building, group dynamics, and interacting with hospital administration skills (2B,3A) |
| 13. Hemmer et al, 2007 | University-based elective program, US | Pathology residents (senior) and fellows | Annual course of 6 sessions (1-2 days each)  ***Credential:***  None specified | - Lectures (didactic) - Seminar (capstone) - Sessions (interactive) - Computer lab instruction - Case scenarios - Teambuilding exercises   ***Guiding frameworks:*** ACGME | - Leadership and management - Change management - Interpersonal skills - Personnel issues - Quality assurance - Informatics - Finance   ***Assessment and Feedback:***  Knowledge tests | 7.5 | 1  2B | ***Methods:*** Pre-post knowledge tests; post-session questionnaires  ***Outcomes:***   - Positive review of program effectiveness (1) - Significant increase in understanding of leadership and management (2B) |
| 14. Kuo et al., 2010 | University-based residency program, US | Pediatric residents  Entry - PGY 1-3 | Integrated into core 3-year residency curriculum  ***Credential:***  None specified | - Seminars (small group) - Experiential rotation (Collaborative child advocacy project) - Advisory groups (Faculty-resident) - Mentorship - Reflection   ***Guiding frameworks:*** Institutional leadership model (Purpose, People, Process, Personal) | - Leadership - Critical thinking - Community engagement including sustainability and communication - Health disparities - Social determinants of health - Health policy - Health economics - Special populations - Project management - Grant writing - Program evaluation - Organizational Structure - Finances/budgeting   ***Assessment and Feedback:***  Peer-feedback  Feedback from program/site staff on leadership interactions | 7.5 | 1,  2B  4 | ***Methods:*** Exit survey; Review of residency projects, and post-graduate achievements – methods not explicit  ***Outcomes:***   - Satisfaction with program and perceived long-term impact on career goals and plans (1) - Significant increase in self-reported competence as a leader and clinical education/skills (2B) - Graduate achievements including presentations, publications, and awards (3B) - Project impacts at program, policy, advocacy levels (e.g., development of new health clinics, increasing staff diversity, championing new legislation) (4) |
| 15. Levine et al., 2008 | University-based elective program, US | Incoming chief residents from multiple specialties (program directors/faculty mentors also invited) | 2-day retreat completed during year as chief resident  ***Credential:***  None specified | - Case-based, small group learning - Seminars - Lectures (mini) - Action projects   ***Guiding frameworks:*** N/A | - Inter-specialty care - Teaching skills (small groups, reluctant learners) - Clinical leadership - Giving feedback - Conflict resolution - Special population care (geriatrics)   ***Assessment and Feedback:***  Pre- and post-MCQ knowledge tests | 10 | 1,  2A,B 3A | ***Methods:*** Mixed-method; Pre-post MCQ tests & surveys; Long-term follow-up interviews  ***Outcomes***:   - Positive review of program effectiveness and perceived positive impact on clinical work (1) - Insignificant increase in tested geriatric knowledge (neutral-2B); Significant increases in self-reported knowledge (2B) - Increased confidence (2A) - Enhanced skills in teaching, clinical teaching (geriatrics), leadership, group facilitation, giving feedback, connecting with reluctant learners, conflict resolution, managing multiple responsibilities, and inter-specialty collaboration (2B) - Improved clinical skills and practice in geriatric care (2B) - Heightened sensitivity to needs of target population (2A) - Improved patience with patients (3A) - Improved communication with patients (3A) |
| 16. LoPresti et al., 2009 | Formal residency program, US | **F**amily medicine residents –  Entry - Year 2 | 1-year simulated practice training curriculum    ***Credential:***  None specified | - Lectures - Projects (individual/ group) - Simulated practice decision-making projects   ***Guiding frameworks:*** N/A | - Marketing (market analysis) - Strategic planning - Office space and design, equipment - Information systems - Policy and procedure manuals - Management (personnel/staffing) - Leadership - Quality Improvement - Scheduling and triage - Insurance contracts   ***Assessment and Feedback:***   - Pre- and post-MCQ and pick-N exams | 12.5 | 2B | **Methods:** Pre-post survey with comparison group  **Outcomes:**   - Significant increases in all areas for residents with simulated practice training (market analysis and strategic planning, office space and design, computer technology, staff hiring and management, leadership, continuous QI, marketing, office scheduling, federal agencies/laws, insurance and personal finance, negotiation and contracting, taxation, coding and billing) - Increased knowledge evidenced by higher final exam scores for intervention group (2B) |
| 17. MacCarrick, 2014 | Training program (developed by national medical college), Australia | Residents and senior physicians in multiple specialties  Entry at Year 4+ | Medical administration curriculum – length varies  ***Credential:***  None specified | - Workshops - Learning sets (group-based; cross-boundary collaboration) - Reflective practice - Coaching/preceptorship - Masters level coursework   ***Guiding frameworks:*** Relational leadership;   - Royal Australasian College of Medical Administrators (RACMA) medical leadership and management framework; | - Health law and ethics - Health economics - Healthcare systems - Financial management in health - Epidemiology and statistics - Leadership - Research skills   ***Assessment and Feedback:***   - Self-discovery inventories (e.g, personality) - College examination - Peer assessment - Annual in-training assessment (supervisor/ preceptor feedback on competency achievement across domains) | N/A | N/A | No evaluation data. provided. |
| 18. Micallef & Straw, 2014 | Training program (coordinated by government agency), Australia | Resident medical officers (  PGY 2 -3) and registrars in multiple specialties | Three 10-11 week rotations over 31 weeks  ***Credential:***  None specified | - Project (service improvement with advisor) - Site visits (industry/health care organizations) - Leadership masterclasses - Workshops - Shadowing leaders (hospital executives)   ***Guiding framework:*** N/A | - Clinical Service Redesign - Management (general) - Leadership styles - Presentation skills - Mindfulness and meditation   ***Assessment and Feedback:***  None specified. | N/A | 2A  3A  4 | ***Methods:*** Focus group mentioned, but no formal evaluation methods provided.  ***Outcomes:***   - Participants were satisfied with program (1) - Perceived positive impacts on career prospects and clarification of career paths (2A) - Increased interdisciplinary networking and relationship building (3A) - Increased understanding of the health system (2B) - Hospital have sought to hire graduates from this program (4) - Improved hospitals processes from service improvement projects (e.g., clinical handover between departments, outpatient clinic waiting times, access to diagnostic tests) (4) - System-level knowledge transfer about service improvement opportunities (i.e., increasing recognition of similar problems and issues across sites has increased dialogue between sites) (4) |
| 19. Moore et al., 2016 | University-based elective program, US | Internal medicine residents – Year 1 | Twelve 90-minute modules over 1 year (18 hrs total; 1 module/month)  ***Credential:***  None specified | - Group discussions (panel/small/large group) - Sessions (interactive/didactic) - Case-based learning   ***Guiding framework:*** N/A | - Leadership in medicine - Leadership vs management - Professionalism - Emotional intelligence - Leadership styles - Authentic leadership - Conflict management - Team decision making and bias - Communication (in multi-disciplinary teams/ with adult learner)s | 6 | 1 | ***Methods:*** Focus groups, pre-program survey  ***Outcomes:***   - Majority of participants recommended program (1) |
| 20. Nabili, 2016 | University-based residency program, US | Surgery residents – level not specified | 2-year contiguous curriculum  ***Credential:***  None specified | - Lectures - Group discussion (interactive, scenario based) - Journaling (daily gratitude)   ***Guiding framework:***  ***Patient-based leadership***  ACGME core competency framework | - Transformational leadership - Credibility - Time management - Emotional intelligence - Conflict resolution - Consciousness in leadership - Appreciative inquiry - Co-leadership (interprofessional – surgeon, nurse) - Personal wellbeing   ***Assessment and Feedback:***   - Exam (post-program) | N/A | N/A | No evaluation data provided. |
| 21. Patel et al., 2015 | Formal integrated residency program,  US | Residents (PGY1-2) in multiple specialties  with interest in healthcare quality/patient safety | 2-year longitudinal training pathway embedded in standard residency program  ***Credential:***  None specified | - Lectures - Online modules - Small-group activities/tutorials - Facilitated discussion) - Experiential placement (short-term interprofessional setting) - Experiential placement (longitudinal) in QI leadership team - Capstone QI project (longitudinal) - Mentorship from multi-group of professional leaders   ***Guiding framework:*** Institutional (UPHS) Blueprint for Quality and Patient Safety | - Quality improvement and measurement - High-value care - Clinical leadership (unit-based teams) - Human factors engineering - Culture of safety   ***Assessment and Feedback:***  QI Knowledge Assessment | 6.5 | 1  2A,B 3B  4 | ***Methods:*** Post-program surveys, knowledge assessment test, Data from curricular projects; Feedback from program/hospital leadership  ***Outcomes:***   - Positive reviews of curriculum by residents (1) - Perceived improvements in ability to plan and lead QI/PS activities, interest in pursuing QI/PS positions in future (2A) - Increased mean knowledge scores from pre to post test (2B) - Increased scholarly productivity (i.e., conference presentations, publications) (3B) - Perceived improvements to organizational understanding of QI and improvements in healthcare delivery through residents’ projects (4) |
| 22. Patterson et al., 2013 | Regional training program, UK | General practice residents – PGY3 | Elective practice-based projects and associated meetings completed during residency tutorial time  ***Credential:***  None specified | - Practice-based project - Group discussion (based on action learning principles) - Reflections (personal online log)   ***Guiding framework:*** Royal College of General Practitioners' competency framework; MLCF | N/A | 5.5 | 1  2A,B | ***Methods:*** Mixed method (Pre-post survey, Focus groups)  ***Outcomes:***   - Positive perception of program structure and value (1) - Increased self-awareness as reported by both residents and program staff (2A) - Decreased self-assessment among residents across domains (not specified) as reported by program staff (2A) - Better understanding of the complexity of managing change in health systems (2A) - Positive shifts in multiple MLCF domains (no specific information provided) (2B) |
| 23. Pettit et al., 2011 | University-based elective program, US | Upcoming chief residents in neurosurgery | Monthly workshops incorporated into 1 year of an existing residency program - 1  ***Credential:***  None specified | - Workshops - Case studies   ***Guiding framework:*** ACGME | - Leadership styles - Conflict management - Communication styles - Effective feedback - Team building - Team leadership - Motivation - Transition from peer to leader   ***Assessment and Feedback:***  Self-assessment | 9 | 2A,B  4 | ***Methods:*** Mixed-method Retrospective pre-post survey with quantitative & qualitative response options; Informal departmental feedback  ***Outcomes:***   - Change in perceptions from seeing leadership as innate to a skill that you can improve (2A) - Significant increase in knowledge about leadership (2B) - Departments report smoother transition of chief residents (4) |
| 24. Runnacles et al., 2013 | Hospital-based elective program, UK | Residents in multiple specialties – level not specified | Level One: introductory workshop (1 day);  Level Two 6 months elective: 2 full-day workshops and eQI project  Level 3 (9 months with experiential placement):  ***Credential***:  None specified | - Workshops - Reflection (group, Project surgeries) - Study days - Online learning group - Project (QI) - Mentorship and coaching - Experiential immersive work placement - Patient safety walk-arounds - Peer-to-peer teaching   ***Guiding framework:*** NHS Leadership competency framework | - Quality Improvement methodology, - Patient safety - Human factors - Clinical leadership - ***Assessment and Feedback:***   Rapid-cycle project feedback with expert and peer feedback | 6.5 | 1  2A,B  3B  4 | ***Methods:*** Mixed-method incl. Pre-post survey; qualitative data (unclear sources)  ***Outcomes:***   - Positive review of program (1) - Significant increase in confidence in using QI methodology tool (2A) - Significant increase in awareness of importance of QI (2A) - Increased knowledge of QI process and QI methodologies and importance of stakeholder engagement (2B) - Scholarly activities (conferences/awards) (3B) - Improvement of system processes via projects (e.g., to reduce waste of disposable medical instruments, introduce system to record calls to respiratory team, reduce rate of prescribing errors) (4)   Barriers to project success were identified including time limitations, difficulty engaging team, change resistant culture etc. |
| 25. Ruston & Tavabie, 2010 | University-based residency program, UK | General practice residents – level not specified | Leadership/QI placement integrated into residency program – 2 days a week for 4 months plus project  ***Credential:***  None specified | - Peer learning sets - Experiential placement - Individual QI projects   ***Guiding framework:*** MLCF | None specified. | N/A | 1  2A,B  3A  4 | ***Methods:*** Qualitative case study (multiple data sources e.g. interviews, reflective diaries, observations, group minutes, project reports)  ***Outcomes:***   - Residents satisfied with program and valued opportunity to see leadership and management in practice (1) - Increased self-awareness, awareness of importance of value alignment (2A) - Perceived enhancement in self-leadership including self-management, self-development, and acting with integrity and team leadership (2B, 3A) - Increased knowledge about organization (2B) - Residents and supervisors reported that residents had improved problem solving, time management, project management, project planning, conducting needs assessments, collecting evidence, networking, communication and delegation skills (2B) - Enhanced development of networks (3A) - 2 of the 3 QI projects have resulted in organizational change (4) - Supervisors reported reduced organizational silos and cultural barriers (4) |
| 26. Saravo et al., 2017 | University-based elective program, Germany | Residents in multiple specialties – PGY1-4 | 4 weeks (2.5 hrs per week)  ***Credential:***  None specified | - Sessions (didactic) - Simulation scenarios with feedback - Practical communication exercises - Reflection   ***Guiding framework:***  Full range leadership model, Transformational and transactional leadership | - Leadership theory - Clinical team leadership - Communication   ***Assessment and Feedback***   - Instructor feedback on simulated leadership performance - Self-assessment | 13.5 | 2B  3B | ***Methods:*** Controlled trial – non-randomized (external & self-report measures)  ***Outcomes:***   - No significant increase in leadership knowledge, but better gains in intervention group (2B) - Significant increase in self-assessed transactional and transformational leadership skills for intervention group (2B) - Significant intervention effect evidenced by improved (externally evaluated) transactional and transformation leadership skill performance (3A) |
| 27. Stergiopoulos et al., 2009 | University-based elective program, Canada | Psychiatry residents –  PGY 2 and 4 | 4 half day workshops over 6 months  ***Credential:***  None specified | - Workshops (didactic and interactive – including think-pair-share; debates, case studies, buzz groups) - Small group learning - ***Guiding framework:*** CanMEDS | - Teamwork - Conflict resolution - Negotiation - Quality Improvement - Program evaluation - Change management leadership - Mental health and addictions reform - Organizational structures in mental health - Career/self-development   ***Assessment and Feedback***  None specified. | N/A | 1 | ***Methods:*** Post-feedback survey. No formal outcomes measured.  ***Outcomes:***   - Positive reviews of program (1) |
| 28. Stoll, 2011 | Fellowship training program (coordinated by government), UK | Trainee doctors – level not specified, multiple specialties | 1 year - out of program specialty training.  ***Credential:***  Accredited postgraduate certificate | - Projects - Coaching - Workshops   ***Guiding framework:*** MLCF | - Service redesign - Quality and Safety Improvement - Leadership capacity building   ***Assessment and Feedback***  None specified. | 8.5 | 2A,B  4 | ***Methods:*** Mixed method (post-program questionnaire, interviews with program staff and participants)  ***Outcomes:***   - Trainees and directors reported growth in trainee self-understanding (2A) - Trainees reported transformation of beliefs and values about the role of clinical leadership and/or inaction as well as revision of career aspirations (2A) - Trainees and directors reported and increased understanding of organizational contexts, enhanced ability in working with others; acquisition of change management and service improvement skills (2B) - Increased stakeholder belief in trainees’ abilities to produce change (4) - Successful trainee projects resulted in tools, and programs that institutions utilized to improve health care outcomes (4) - Organization capacity building through greater engagement of trainees in service improvement activities (4) |
| 29. Stretz et al. | Training program (delivered by medical society), US | Medical students, residents, fellows | Monthly meetings – 6 months  ***Credential:***  None specified | - Projects (team-based) - Lectures (didactic) - Small group exercises - Mentorship   ***Guiding framework:***  ACGME | - Effective leadership - Networking skills - Organizational behaviour - Negotiation skills - Executive decision making - Emotional intelligence - Financing health care - Alternative payment models   ***Assessment and Feedback***   - Leadership style and emotional intelligence inventories with feedback | 6 | 1  2A 3A | ***Methods:*** Pre-post survey  ***Outcomes:***   - Program met participant expectations (1) - Increased comfort in ability to navigate, interact, and influence governmental actors to effect cultural, structural, and policy change (2A) - Increased sense of comfort with communication skills to effect change (2A) - Increased experience with leadership (3A) - *Program maintained diversity – increasing overall representation of minority groups (Asian/African American) from pre-completion to post-completion (decline in Latino participation) - *Fewer women than men dropped out of the program |
| 30. Warren et al. | Training program (coordinated by government), UK | Medical registrars - specialist and GP – level not specified | Formalized mentoring relationship with senior healthcare leader – 1 year  ***Credential:***  None specified | - Mentorship (formalized - central element) - Workshops/seminars - Experiential learning/placement - Leadership masterclass   ***Guiding framework:***  MLCF; Leadership Qualities Framework; Leadership for London framework | - Power, politics, and persuasion - Patient safety - Workforce development in public/private sectors - Policy development - Service reconfiguration - Leadership and strategy - Leading in the NHS   ***Assessment and Feedback***  None specified. | N/A | 1  2A 3B | ***Methods:*** Feedback event. No formal evaluation methods  ***Outcomes:***   - Positive feedback from mentors and mentees about mentoring relationships and seminars (1) - Increased confidence and interest in pursuing clinical leadership roles (2A) - Mentees appointed to various leadership positions and roles (3B) |
| 31. Voogt et al. | University-based elective program, the Netherlands | Residents – PGY 1 – 6 | ***Credential:***  None specified | - Group QI projects - Discussion   ***Guiding framework:***  Integrated model of behaviour prediction; self-determination theory | - QI   ***Assessment and Feedback***   - Feedback from program staff | N/A | 1  2A, B | ***Methods:*** Interviews, observations of meetings, document analysis  ***Outcomes:***   - Residents felt heard and taken seriously (1) - Residents reported increased self-awareness and of organizational aspects of health care delivery (2A) - Residents reported increased ability to identify relevant stakeholders, barriers, and facilitators of successful QI (2B) |

1. Kirkpatrick level outcomes [↑](#footnote-ref-1)
2. Medical Leadership Competency Framework [↑](#footnote-ref-2)
3. ACGME = Accreditation Council of General Medical Education [↑](#footnote-ref-3)
