# Supplementary material for: Is postgraduate leadership education a match for the wicked problems of health systems leadership? A critical systematic review
Source: Perspect Med Educ. 2019 Jun 3;8(3):133–42. doi: 10.1007/s40037-019-0517-2 (PMC6565666; doi:10.1007/s40037-019-0517-2)
Supplement: Supplementary file 3 — Summary of postgraduate leadership programs’ guiding frameworks, curricular content, instructional approaches, and evaluation designs (N = 31) [file 40037_2019_517_MOESM3_ESM.docx]

| Table 3. *Summary of postgraduate leadership programs’ guiding frameworks, curricular content, instructional approaches, and evaluation designs (N = 31)* |
| --- |

| **Program feature** | **No. (%)** |
| --- | --- |
| **Guiding leadership frameworks and/or theories informing program design**   - ACGME competencies^8,56,81,82,30^ - Adaptive leadership and collective leadership^33^ - CanMEDS^48^ - Emotional intelligence theory/models^36,38^ - Full range leadership model (transformational leadership, etc.)^35^ - Local institutional/organizational model^40,44^ - MLCF^47,50,51^ - MLCF, Leadership Qualities Framework, and Leadership for London framework^23^ - MLCF and Shared Leadership^32^ - MLCF and Royal College of General Practitioners' Framework^45^ - NHS Leadership Competency Framework^46^ - RACMA and relational leadership model^34^ - US military leadership curriculum^37^ - Integrated model of behaviour prediction and self-determination theory^49^ - None specified^2,41–43,52–55,76^ | 5 (16.1)  1 (3.2)  1 (3.2)  2 (6.5)  1 (3.2)  2 (6.5)  3 (9.8)  1 (3.2)  1 (3.2)  1 (3.2)  1 (3.2)  1 (3.2)  1 (3.2)  1 (3.2)  9 (32.5) |
| **Teaching methods**^1^   - Case studies/scenarios or case-based learning^2,8,39,41,43,52,53^ - Coaching^33,34,46,50,56^ - Computer lab instruction^39^ - Courses (masters level)^33,56^ - Experiential rotations (practicum/placement) ^23,33,37,40,44,46,51,56,76^ - Group discussions^2,36,43–45,49,52,55,82^ - Inter-specialty care^41^ - Journal club^56^ - Journaling^82^ - Leadership masterclass^23^ - Learning sets (action/group-based)^32,34,47^ - Lectures/workshops/seminars/sessions (didactic/with interactive components^8,23,33–36,38,40–44,46,48,50–54,56,76,81,82^ - Leadership masterclasses^42^ - Mentorship^23,40,44,46,56,76,81^ | 7 (22.6)  5 (16.1)  1 (3.2)  2 (6.5)  9 (29.0)  9 (29.0)  1 (3.2)  1 (3.2)  1 (3.2)  1 (3.2)  3 (10)  23 (9.7)  1 (3.2)  7 (22.6) |
| - Online learning (discussion/portfolio/)^38,44,46,51,56^ - Peer-to-peer teaching^46^ - Personal study^32,46,55^ - Projects (individual/group/action) ^32,41,42,44–47,49,50,54,55,76,81^ | 5 (16.1)  1 (3.2)  3 (9.7)  13 (41.9) |
| - Reflection^32,34,35,40,45,46^ - Role play^2,51,53^ - Rounds (walk-arounds, work-rounds)^46,56^ - Shadowing^32,42^ | 6 (19.4)  3 (9.7)  2 (6.5)  2 (6.5) |
| - Simulation (scenario models)^35,53,54^ | 3 (9.7) |
| - Site visits^42^ - Small group learning (exercises/tutorials)^2,44,48,52,53,81^ - Team building exercises^39^ | 1 (3.5)  6 (19.4)  1 (3.2) |
|  |  |
| **Content areas^2^** |  |
| - Adaptive leadership competencies^33^ - Appreciative inquiry^82^ - Assertiveness^51^ - Authentic leadership^2,43^ - Business management (e.g., specialty specific)^51,55^ - Career development^48^ - Chairing meetings^51^ - Change management (change implementation, service reconfiguration)^23,33,38,39,48^ - Clinical leadership (clinical team leadership, clinical enterprise management)^2,35,41,44,46,76^ - Clinical microsystems (service design, improvement)^33,42,50^ - Co-leadership (interprofessional)^82^ - Collaboration and cooperation^37^ - Communication^8,35,36,38,43^ - Community engagement (sustainability, communication)^40^ - Conflict management/resolution^8,33,36–38,41,43,48,52,53,82^ - Consciousness in leadership^82^ - Credibility^82^ - Critical thinking^40^ - Cultural competence^38^ - Decision making^37,81^ - Delegation^53^ - Difficult conversations^51^ - Economics (healthcare)^34,40,52,55^ - Emotional intelligence^2,36,38,43,81,82^ (emotional awareness and reframing)^33^ | 1 (3.3)  1 (3.2)  1 (3.2)  2 (6.5)  2 (6.5)  1 (3.2)  1 (3.2)  5 (16.1)  6 (19.4)  3 (9.7)  1 (3.2)  1 (3.2)  5 (16.1)  1 (3.2)  11 (35.5)  1 (3.2)  1 (3.2)  1 (3.2)  1 (3.2)  2 (6.5)  1 (3.2)  1 (3.2)  4 (12.9)  7 (22.6) |
| - Epidemiology and statistics^34^ - Equity (health disparities)^40^ - Global strategy and program development ^76^ - Governance and performance^32^ - Group governance and mergers^52^ - Feedback (delivery)^8,36,41,53^ - Financial management and planning (budgeting, costing analysis)^33,34,39,40,52,53,76,81^ - Health systems leadership/management (operations/structure/ systems thinking/public leadership)^32–34,38,51,76^ - Hedging risk ^53^ - High-value care^44^ - Human factors^37,44,46^ - Information technology/systems/Informatics^39,54,76^ - Insurance contracts^54^ - Interpersonal skills^36,39^ - Law and ethics (health care)^34^ | 1 (3.2)  1 (3.2)  1 (3.2)  1 (3.2)  1 (3.2)  4 (12.9)  8 (25.8)  6 (19.4)  1 (3.2)  1 (3.2)  3 (9.7)  3 (9.7)  1 (3.2)  2 (6.5)  1 (3.2) |
| - Leadership (general)^2,23,34,36,38–40,43,54,81^ - Leadership capacity building^50^ - Leadership vs management^43^ - Leadership role transition^8^ - Leadership styles^2,8,42,43^ - Leadership theory (broad, ^35^ situational leadership^33^, transformational leadership^82^) - Learning (learning organizations, learning on services) ^38,53^ - Management (general/others/performance/staffing/personnel issues/workforce development)^23,39,42,51,54^ - Marketing (market analysis)^54^ - Medical culture^56^ - Motivation (motivation theory, engaging others)^8,51^ - Narrative^33^ - Negotiation^33,48,52,53,81^ - Networking^81^ - Office space design and equipment^54^ - Organizational culture, behaviour, structure, and models^37,38,40,48,81^ - Organizational development and leadership^32,55^ - Personality types^33^ | 10 (32.3)  1 (3.2)  1 (3.2)  1 (3.2)  4 (12.9)  3 (9.7)  2 (6.5)  5 (16.1)  1 (3.2)  1 (3.2)  2 (6.5)  1 (3.2)  5 (16.1)  1 (3.2)  1 (3.2)  5 (16.1)  2 (6.5)  1 (3.2) |
| - Physician/surgeon health and wellbeing (stress management, mindfulness, meditation)^38,42,53^ - Policy (health care/public)^23,32,40,52,54,55^ - Power (positive power and influence, power, politics, and persuasion)^23,33^ - Practice management ( groups and compensation, private practice)^52,53,55^ - Preventive medicine^56^ - Professionalism^38,43^ - Program evaluation^40,48^ - Program planning and design^37^ - Project management^40^ - Public health (local systems, critical issues)^56^ - Quality Improvement and/or Patient Safety (QI methods/statistics, service redesign)^23,33,37–39,44,46,48–52,54,56,76^ - Research enterprise management ^76^ - Research skills^34^ (writing for publication^56^ grant writing^40^) - Service management (scheduling/triage)^51,54^ - Social determinants of health^40^ - Special populations (individual topics)^40,41,48^ - Strategic planning/management^23,33,54^ - Supply chain management^76^ - Teaching skills^41,53^ - Team performance (team work/team building/team decision-making)^8,33,38,43,48,53^ - Technical planning^37^ - Time management^38,51,53,82^ - Values-based health care^38^ | 3 (9.7)  6 (19.4)  2 (6.5)  3 (9.7)  1 (3.2)  2 (6.5)  2 (6.5)  1 (3.2)  1 (3.2)  1 (3.2)  15 (48.4)  1 (3.2)  3 (9.7)  2 (6.5)  1 (3.2)  3 (9.7)  3 (9.7)  1 (3.2)  2 (6.5)  6 (19.7)  1 (3.2)  4 (12.9)  1 (3.2) |
| **Assessment and Feedback^3^**   - Feedback on reflection tools ^32^ - Feedback from peers^32,34,40,46,56^ - Feedback from program staff (or experiential site staff) on role/project performance^33,34,37,40,46,49,51,56^ - Feedback/assessment of simulated activities^35,53^ - Knowledge test/exam^34,35,39,41,43,44,52,54,82^ - Leadership/personality/self-discovery inventories^34,36,81^ - Objective Structured Teaching Encounters^36^ - Self-assessment^8,35,37,56^ - None/Not specified^6,2,38,76,24,28^ | 1 (3.2)  5 (16.1)  8 (25.8)  2 (6.5)  9 (29.0)  3 (9.7)  1 (3.2)  4 (12.9)  9 (29.0) |
| **Evaluation design**   - Quantitative – pre/post survey or knowledge test^38,39,52,53,81^ - Quantitative – pre/post survey with comparison group^54^ - Post program survey ^40,48,55^ - Quantitative – RCT^35^ - Qualitative ^32,43,47^ - Mixed methods^2,8,37,41,44–46,49,50^ - Time series examination with post-program suvey^36^ - Unclear or not specified but outcomes reported ^23,40,42,51,56^ - No evaluation conducted ^33,34,76,82^ | 5 (16.1)  1 (3.2)  3 (9.7)  1 (3.2)  3 (9.7)  9 (29.0)  1 (3.2)  5 (16.1)  4 (12.9) |

^1-3^ Programs can be categorized under multiple teaching methods, content areas, and assessment methods; total % for these categories exceeds 100
